# Extracranial glioblastoma with synchronous metastases in the lung, pulmonary lymph nodes, vertebrae, cervical muscles and epidural space in a young patient - case report and review of literature

**DOI:** 10.1186/1756-0500-6-290

**Published:** 2013-07-25

**Authors:** Christian Blume, Marec von Lehe, Frank van Landeghem, Susanne Greschus, Jan Boström

**Affiliations:** 1 Department of Neurosurgery, University of Bonn Medical Center, Sigmund-Freud-Str. 25, Bonn 53105, Germany; 2 Department of Neuropathology, University of Bonn Medical Center, Sigmund-Freud-Str. 25, Bonn 53105, Germany; 3 Department of Radiology, University of Bonn Medical Center, Sigmund-Freud-Str. 25, Bonn 53105, Germany; 4 Department of Radiosurgery and Stereotactic Radiotherapy, Mediclin Robert Janker Clinic, Villenstrasse 8, Bonn 53129, Germany

**Keywords:** Glioblastoma, Extraneural metastases, Case report

## Abstract

**Background:**

Extraneural and extracranial metastases of glioblastoma (GB) are very rarely reported in the literature. They occur in only 0.2% of all GB patients.

**Case presentation:**

We present a 40 year old caucasian male with secondary GB and first diagnosis of an astrocytoma world health organisation (WHO) grade II through stereotactic biopsy in 2006. He presented a new hemiparesis and a progress of the known mass lesion in 2008. Subtotal tumor resection was performed and the histological examination verified a GB. After combined radio- and chemotherapy the adjuvant temozolomide therapy was not started because of non-compliance.

In 2011 a second local relapse was resected and 4 month later the patient presented a fast progressing tetraparesis. Cervical CT and MRI scan showed a mass lesion infiltrating the fifth and sixth vertebra with infiltration of the spinal canal and large paravertebral tumor masses. Emergency surgery was performed. By additional screening further metastases were detected in the thoracal and lumbal spine and surprisingly also in the lung and pulmonary lymphnodes. Palliative radio- and chemotherapy of the pulmonal lesions was completed, further antitumor therapy was rejected. The patient died 10 months after diagnosis of the extraneural metastases.

**Conclusion:**

Especially young “long-term-survivors” seem to have a higher risk of extraneural metastasis from a GB and appropriate staging should be performed in these cases.

## Background

Glioblastoma (GB) is known as a highly aggressive neuroepithelial tumor almost exclusively growing in the neural tissue. Extraneural metastases were rarely reported in the literature. The incidence of extraneural metastases is 0.2% of all GB patients (Hsu E, 1998) [1]. In 2008 Piccirilli et al. [2] reviewed the literature from 1928 to 2006 and reported about 128 cases of extraneural metastases of GB. Remarkably, the average age of these patients at presentation was 40, whereas otherwise the mean age of the first diagnosis of cerebral GB is 54 years [3]. In addition, the mean overall survival of patients with extraneural metastasis was at least 17 months (Piccirilli et al., 2008). From this, one can conclude that - due to the younger age which is related with better overall survival in GB patients - those patients have the potential developing extraneural metastases.

We present a case of a 40 year old patient with first diagnosis of an Astrocytoma World Health Organization (WHO) grade II in 2006, a secondary GB in 2008 and multiple extraneural metastases in 2011. Although several cases of extraneural metastases of GB have been reported, to our best knowledge this is the first report of a case with so many metastases in different tissues (see Table 1).

**Table 1:** Summarizing publications on GBs with extraneural and extracranial spread

**Author**	**Journal**/**year published**	**Case No.**	**Sex female**/**male**	**Extracranial localisation**					
				***Osseous***	***Spine***	***Lung*** (***incl***. ***Pleura***)	***Liver***	***Lymph nodes***	***Abdominal***
Piccirilli M et al. (review)	Tumori/2008	128	41/82	26	32	51	20	42	14
Templeton A et al.	Onkologie/2008	2	1/1	1	0	2	0	0	0
Rajagopalin V et al. (review)	J Neurooncol/2005	15	unknown	3	12	0	0	0	0
Hsu E et al. (review)	J Neurooncol/1998	1	unknown	1	0	0	0	0	0
Futoshi M et al.	Clin Imag/1994	1	1/0	0	1	0	0	0	0
Beauchesne P et al.	J Neurosurg/1993	1	0/1	0	1	0	0	0	0
Liwnicz H et al.	Hum Pathol/1979	3	0/3	0	2	1	0	0	0
Terheggen HG et al.	Eur J Pediatr/1977	1	0/1	1	0	0	0	0	0
Leifer D et al.	J Neurosurg/1971	1	0/1	0	0	1	0	0	0

## Case presentation

**Figure 1:**
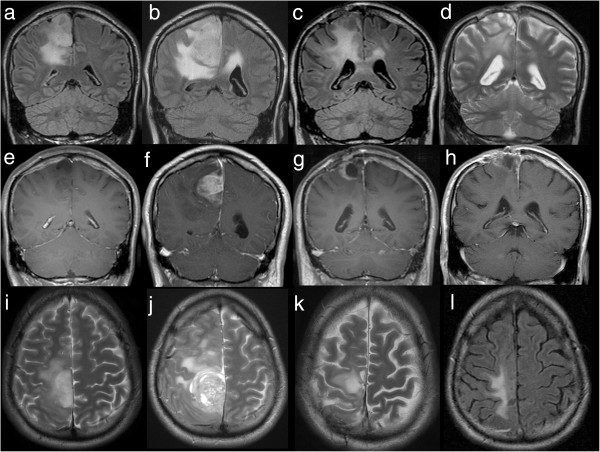
**MRI of the brain at four timepoints.** Each row resembles one examination with coronal acquisition of Flair (or T2, d), coronal contrast enhanced T1 and axial T2 (or Flair,l). After the first MRI in 2006 **(a,e,i)**, showing a non-enhancing tumor, the patient underwent surgery and Astrocytoma WHO II° was diagnosed. In 2008, recurrent tumor **(b,f,j)** with striking contrast enhancement **(f)**, correlating with histologically proven malignisation to Glioblastoma WHO IV°, was proven by MRI. Again tumor recurrence with rim enhancing tumor **(c, g, k)**, was documented in 04/2011 and surgery was performed. In 08/2011 **(d**,**h**,**l)** MRI of the brain showed that the local contrast enhancing tumor was under control and not responsible for the deteriorating neurological status.

At age 35 this caucasian male initially presented with a simple partial seizure on the left side in 2006. There were no other diseases reported in the medical history of the patient. Karnofsky Performance Status (KPS) on admission was 90 and his antiepileptic drug therapy was based on oxcarbazepine and clobazam. Magnetic resonance imaging (MRI) showed a mass lesion (3 centimeter (cm) × 2 cm) in the right central region (see Figure 1).

Stereotactic biopsy histologically verified an Astrocytoma WHO grade II. Postoperatively the patient suffered a mild hemiparesis on the left side, which regressed on dexamethason. Because of the histological result no adjuvant therapy was performed, only regular MRI controls were planned.

In 2008 the patient presented an aggravated hemiparesis, a new hemihypaesthesia and a coordination failure on the left side. The patient reported about an increase of the seizure frequency with a simple partial seizure every four days. Follow up MRI showed a progress of the known mass lesion with new contrast medium enhancement (see Figure 1).

A debulking with subtotal tumor resection was performed and the histological examination verified a secondary glioblastoma multiforme (GB). After the surgery a combined radiation therapy (RT) (5 × 2 Gray (Gy) per week, ad total dose of 60 Gy) and chemotherapy (75 mg/m^2^ temozolomide per day) was performed, but because of non-compliance the planned adjuvant temozolomide chemotherapy was not started.

Because of recurrent tumor growth and chronic local wound infection a revision surgery was performed in April 2011 (see Figure 1).

The histology showed recurrent glioblastoma and microbiologically propionibacterium was isolated and treated with metronidazole and flucloxacillin. Clinically the patient remains stable with a mild hemiparesis left and a KPS of 70 at discharge.

**Figure 2:**
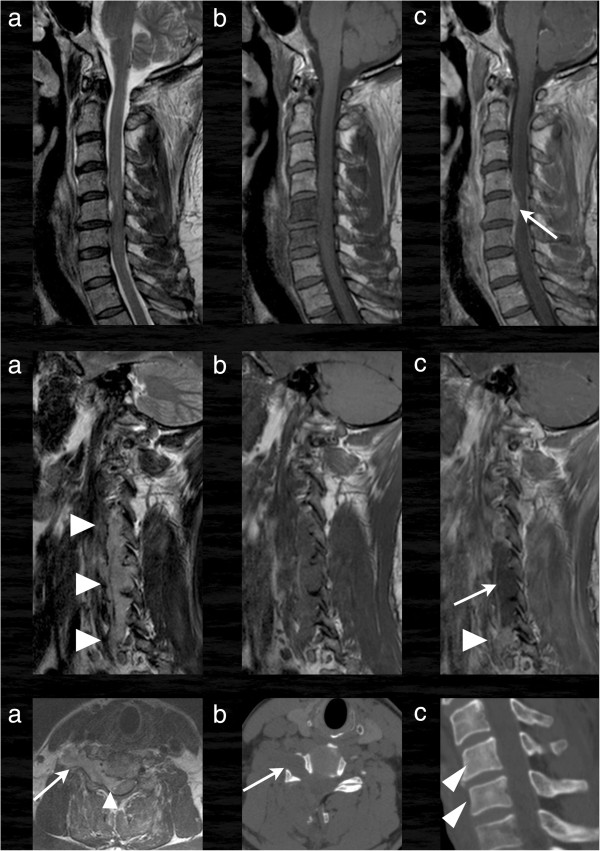
**Cervical MRI and CT.** In the first and second row sagittal and parasagittal images of the cervical spine in T2 **(a)**, T1 **(b)** and T1 with contrast enhancement **(c)** are shown. There is a vertebral tumor infiltration of the fifth and sixth vertebra with a small invasion into the spinal canal (arrow). Larger tumor masses are found in the right lateral paravertebral space (second row). The tumor is hyperintens in T2 **(a, arrowheads)**, hypointens in T1 and is partially contrast enhancing (arrowhead) with central non-enhancing parts **(arrow in c)**. Remarkably the tumor grows through the intervertebral foramina in an hour glass pattern **(last row, a)** like a schwannoma even with enlargement of the osseous canal **(arrow in b, axial CT)**. The infiltrated vertebrae are only slightly hyperdens compared to the normal vertebrae **(arrowheads in c)**.

In August 2011 the patient was admitted to the neurosurgical department again because of a fast progressing tetraparesis and ischuria over a few days. The tetraparesis was accentuated on the left side. KPS was 30 at this time. MRI of the brain showed that the local contrast enhancing tumor was under control and not responsible for the deteriorating neurological status. CT and MRI scan of the cervical spine revealed a tumor infiltration of the fifth and sixth vertebra with an invasion into the spinal canal and large paravertebral tumor masses (see Figure 2).

**Figure 3:**
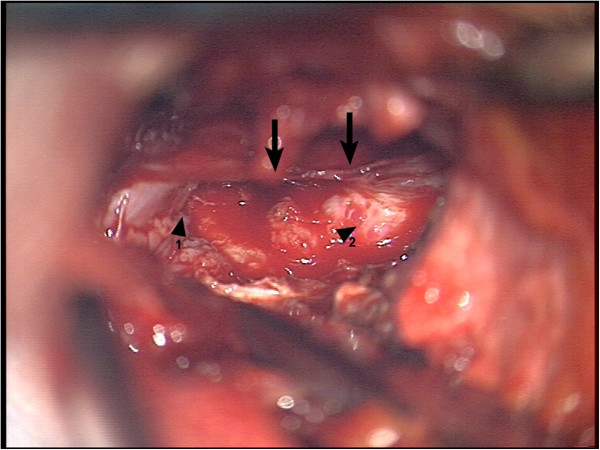
**Intraoperative view 1.** First step of decompression through laminectomie of C5. Arrows show the spinal cord. Arrowhead 1 shows extraspinal tumormass infiltrating paravertebral muscles. Arrowhead 2 shows intraspinal/extradural tumormass.

**Figure 4:**
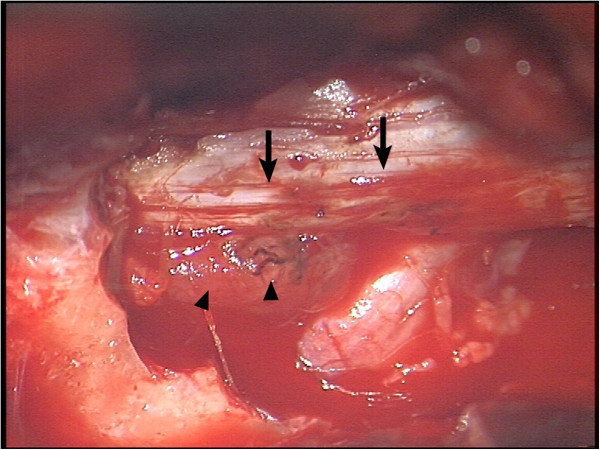
**Intraoperative view 2.** Segment C5 after bony decompression. Arrows point on spinal cord. Arrowheads show the intraspinal tumormass with GB typical necrosis and compression of the myelon.

Emergency surgery on the cervical spine with laminectomy of cervical vertebra 5 and 6, pediculotomy of cervical vertebra 6 and subtotal tumor resection was performed (see Figures 3 and 4).

Additionally a biopsy of the vertebral body of C 6 was taken.

**Figure 5:**
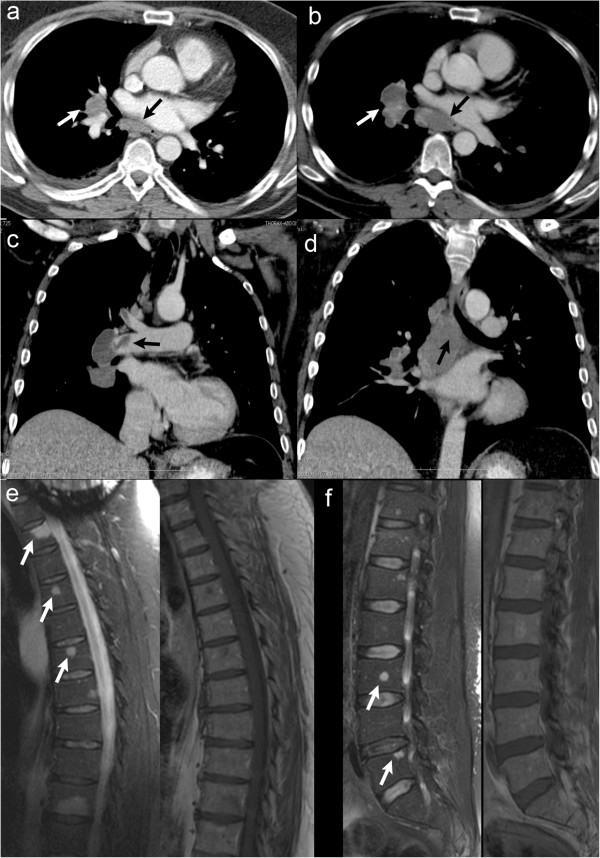
**Staging with CT of the thorax and spinal MRI.** Contrast enhanced CT of the thorax **(a**-**d)** revealed rapidly growing **(a in 05/2011, b-d in 08/2011)** hilar **(white arrow in a and b)** and infracarinal **(black arrow in a,b,d)** lymph node metastases with infiltration of the right pulmonary artery. Additionally thromboembolic material was found in branches of both pulmonary arteries **(arrow in c)**. MRI of the thoracic **(e)** and lumbar **(f)** spine showed multifocal osseous metastases (arrows).

**Figure 6:**
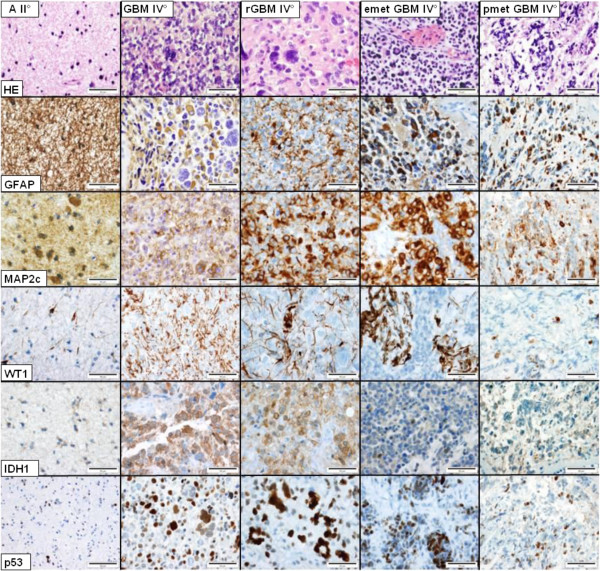
**Neuropathological results.** In the astrocytoma WHO grade II (A II°, left row) density and pleomorphy of the tumor cells are low to moderately pronounced. Tumor cells express GFAP, MAP2c, WT1 and mutated IDH1 (R132H). A subpopulation of ca. 1-2% shows a nuclear accumulation of p53. The glioblastoma multiforme WHO grade IV (GB IV°) has a markedly increased density and pleomorphy, combined with an increased mitotic and proliferative activity, necrotic areas and microvascular proliferations. Compared to the primary astrocytoma, GFAP expression is present only in a subpopulation of tumor cells (ca. 65%) whereas nuclear accumulation of p53 occurs in more tumor cells (5-10%). In the relapsed glioblastoma (rGB IV°) the percentage of tumor cells expressing GFAP (ca. 30%) and WT1 (ca. 10%) decreases. Both, the epidural (emetGB IV°) as well as the pulmonal (pmetGB IV°) metastasis demonstrate expression of GFAP in 10-20% and of WT1 only in small groups and single tumor cells (<10%). Bars = 50 μm, corresponding to a magnification ×200.

In suspicion of a pulmonary embolism after surgery a CT-scan of the thorax was made and, surprisingly, extended right hilar and infracarinal tumor masses with infiltration of the right pulmonary artery were detected. Additionally thromboembolic material was found in branches of both pulmonary arteries (see Figure 5). The mass lesions in the lung and the cervical spine were histologically confirmed as metastases from the cerebral glioblastoma (see Figure 6).

Because of the multiple mass lesions in different tissues staging was completed with MRI of the whole spine showing multifocal osseous metastases (see Figure 5). Lumbar puncture was made with no pathological result. The abdomen CT showed no pathological findings. Postoperativly the tetraparesis regressed, lung function was normal and KPS on discharge was 40.

A combined RT and chemotherapy was subsequently performed in September 2011 with irradiation of the metastatic mediastinal, infracarinal, pretracheal, retrocaval and hilar lesions in a fractionation of 15 × 3 Gy up to a total target volume dose of 45 Gy in a palliative intention. RT was well tolerated without significant acute radiation-induced toxicities. Additional chemotherapy with temozolomide (150 mg/m^2^, 300 mg absolute per 5 days), was performed once, a repetition was again rejected by the patient.

The patient died in June 2012, 10 months after the last RT and chemotherapy.

## Conclusions

The first reported case of extraneural GB metastases was in 1928 [4]. In 2008 Piccirilli et al. [2] have collected about 128 cases of extraneural metastases of GB. Metastases in many different regions of the body in our patient with a secondary GB, especially the combination of lung and osseous lesions with intraspinal involvement makes this new case in many aspects unique. Most of the patients with extraneural GB lesions had metastases in the lung (n = 44), osseous metastases occured as well (n = 29), but there was only one other patient reported with an intraspinal extradural metastasis [2], whereas GB metastases are well known to spread through the cerebrospinal fluid (CSF), as drop metastases [5,6].

Nearly all (96%) reported patients with extraneural metastases underwent cranial GB surgery beforehand (Huang et al., 1995) [7], therefore the haematogenous spreading of GB cells intraoperatively seems to be most likely (Terheggen und Muller, 1977) [8]. Only very rarely, GB patients treated without any surgery developed extraneural metastases [9].

Another special feature in our patient was the fact that the last tumor resection surgery was at the same time a revision for a chronic wound healing disturbance.

By enhancing global immune responses patients with wound infections after GB resection are discussed to have a longer overall survival compared to patients without wound infections, although there is still no clear proof for this theory (De Bonis et al., 2011) [10]. The combination of wound infection and tumor resection brings up the hypothesis that the chronic wound infection in our case perhaps allowed on the one hand a longer survival as usual - with the increased possibility for developing extraneural metastasis - and on the other hand the wound revision procedure itself maybe enabled the haematogenous pathway for metastatic spread. Alternatively, one might suspect that the chronic inflammation in our case had opened the blood–brain barrier and enabled the highly unusual extraneural and extracranial metastatic spread in our case.

The fact that the overall survival in patients with GB increased in the last decade [3] brings up the thoughts if there shouldn’t be a more intensive focus on an appropriate staging for “long-term survivors” with GB.

We can reaffirm with our case that especially young glioblastoma patients have the potential to develop extraneural metastasis. We conclude that especially in the case of the so called “long-term survivors” with repeated surgeries the possibility of extraneural metastasis should be anticipated and an appropriate staging should be performed early when suspected.

Based on the experience with our special case and according to the current literature, we recommend appropriate routine staging consisting of holospinal MRI and CT of thorax and abdomen beside the routine cranial MRI for “long-term survivors” with GB in case of clinical suspicion of extraneural metastasis.

## Consent

Written informed consent was obtained from the next of kin of the patient for publication of this case report and any accompanying images. A copy of the written consent is available for review by the editor of this journal.

## Abbreviations

MRI: Magnetic resonance imaging; CT: Computed tomogram; RT: Radiotherapy; Gy: Gray; KPS: Karnofsky performance index; GB: Glioblastoma; WHO: World health organization; cm: Centimeter; CSF: Cerebrospinal fluid; GFAP: Glial fibrillary acidic protein; IDH: Isocitrate dehydrogenase; MAP: Microtubule-associated protein.

## Competing interests

The authors declare that they have no competing interests.

## Authors’ contributions

FvL contributed and listed up the different histological figures. SG contributed and listed up the radiological figures. MvL performed surgery and contributed the intraoperative pictures. JB accompanied the post surgical therapy of the patient in terms of RT and chemotherapy and as senior author participated significantly in the drafting. CB conceived and drafted the case report as main author. All authors read and approved the final manuscript.
